# Knowledge of tuberculosis (TB) and human immunodeficiency virus (HIV) and perception about provider initiated HIV testing and counselling among TB patients attending health facilities in Harar town, Eastern Ethiopia

**DOI:** 10.1186/1471-2458-13-124

**Published:** 2013-02-08

**Authors:** Ayichew Seyoum, Mengistu Legesse

**Affiliations:** 1 Collage of Medical Sciences, Haramaya University, P.O. Box 235, Harar, Ethiopia; 2 Aklilu Lemma Institute of Pathobiology, Addis Ababa University, P.O. Box 1176, Addis Ababa, Ethiopia

## Abstract

**Background:**

Tuberculosis (TB) and Human Immunodeficiency Virus (HIV) co-infection is one of the major health problems in Ethiopia. The national TB and HIV control guideline in Ethiopia recommends provider initiated HIV testing and counselling (PITC) as a routine care for TB patients. However, the impact of this approach on the treatment seeking of TB patients has not been well studied. In this study, we assessed knowledge of TB and HIV, and perception about PITC among TB patients attending health facilities in Harar town, Eastern Ethiopia.

**Methods:**

In a health facilities based cross-sectional study, a total of 415 study participants were interviewed about knowledge of TB and HIV as well as the impact of HIV testing on their treatment seeking behavior using a semi-structured questionnaires.

**Results:**

Multivariable logistic regression analysis showed the association of distance > 10 km from health facility [adjusted odds ratio (AOR)=0.48, 95% CI: 0.24 - 0.97, P=0.042] with low knowledge of TB. Distance > 10 km from health facility (AOR= 0.12, 95% CI: 0.06 -0.23, P < 0.001) was also associated with low knowledge of HIV testing. Delay in treatment seeking was associated with female participants (AOR = 0.11, 95% CI: 0.05-0.25, <0.001), single marital status (AOR =0.001, 95% CI: 0.00 - 0.01, P< 0.001) and distance > 10 km from health facility (AOR =0.46, 95% CI: 0.28 - 0.75, P=0.002). Most of the study participants (70%) believed that there is no association between TB and HIV/AIDS. On the other hand, two thirds (66.5%) of the participants thought that HIV testing has importance for TB patients. However, the majority (81.6%) of the study participants in the age category less than 21 years believed that fear of PITC could cause delay in treatment seeking.

**Conclusion:**

The study showed the association of low knowledge of the study participants about TB and HIV testing with distance > 10 km from health facility. Study participants in the age category less than 21 years thought that fear of PITC could cause treatment delay of TB patients. Hence, emphasis should be given to improve knowledge of TB and HIV among residents far away from health facility, and attention also needs to be given to improve the perception of individuals in the age group less than 21 years about PITC in the present study area.

## Background

Tuberculosis (TB) is a deadly infectious disease caused by various species of *Mycobacteria*, mainly *Mycobacterium tuberculosis* (Mtb). In 2010, there were 8.8 million (range, 8.5-9.2 million) incident cases of TB, 1.1 million (range, 0.9-1.2 million) deaths from TB among Human Immunodeficiency Virus (HIV) negative people and an additional 0.35 million (range, 0.32-0.39 million) deaths from HIV-associated TB globally [1]. However, the proportion (82%) of TB cases co-infected with HIV is highest in countries in the African Region [1]. Moreover, it is estimated that 50 to 60% of HIV infected people will develop TB disease in their lifetime in contrast with HIV negative persons, whose lifetime risk is only 10% [2].

Ethiopia is among the 22 TB-high burden countries (HBCs) in the world that account for over 80% of the world’s new TB cases each year. During the year 2011, a total of 159,017 TB cases were notified in Ethiopia. Among these, 154396 (97.1%) were new cases of all forms of TB. The proportion of new smear-positive, smear negative and extra pulmonary TB (EPTB) among all new TB cases were 32%, 34%, and 32% respectively [3]. Ethiopia is also among the 41 high TB/HIV burden countries. In the year 2011, there were about 38000 HIV-positive new TB cases in the country. Among 65140 TB patients who were screened for HIV infection, 5,442 were found to be positive for HIV infection [3]. The problem of TB is attributed to a number of factors like a large number of undetected and untreated infectious TB cases, poverty, high prevalence of HIV and low health facilities in rural areas [4-7].

The WHO recommends HIV testing among all TB patients, provision of co-trimoxazole preventive therapy and antiretroviral therapy for HIV-positive TB patients, intensified case-finding for TB among people receiving HIV care and isoniazid preventive therapy for HIV-positive people without active TB [1]. This would be practical only if all TB patients volunteer to be tested for HIV.

The rate of TB HIV co-infection is high in Ethiopia ranging from 25% to 57% in different regions of the country [8,9]. Thus, the national TB and HIV control guideline in Ethiopia recommends provider initiated HIV testing and counselling (PITC) as a routine care for all TB patients [10]. However, there is little information on the effect of this approach on the treatment seeking of TB patients [11]. A better understanding about the barriers to the treatment seeking of TB patients is important to identify effective strategies to improve treatment seeking among TB suspects. Therefore, the aim of this study was to assess the knowledge of TB and HIV as well as perception about PITC among TB patients attending health facilities in Harar town, Eastern Ethiopia.

## Methods

### Study area and population

Between March and May 2011, a cross-sectional study was undertaken to assess the knowledge of TB and HIV, as well as the perception of PITC among TB patients attending health facilities in Harar town, Eastern Ethiopia. Based on the 2007 census conducted by the Central Statistical Agency (CSA) of Ethiopia [12], Harar has a total population of 183,344. Health infrastructure of the region is served by 4 hospitals, 10 health centers and 20 Health posts. Treatment for TB is given freely in all governmental health institutions, but antiretroviral therapy for HIV patients is given only in governmental hospitals and in two health centers located in the town. Based on the accessibility for patients and availability of directly observed treatment, short course (DOTS) and HIV testing, Harar Hiwot Fana, Jegula and Harar federal police hospital that with average 10 TB patients visit per day were purposively selected for this study.

#### Data collection

All TB patients attending the selected health facilities during the study period, and who met the eligibility criteria (age above 15 years, non-pregnant women, patients who were not critical ill, patients who had no problem of hearing or speaking) and volunteer to participate in the study were interviewed about TB, HIV, HIV testing and their treatment seeking using a semi-structured questionnaires. The questionnaires consisted of questions related to socio-demographic characteristics, patient’s knowledge about TB, HIV, TB/HIV association and HIV testing and their treatment seeking behavior. The interview was done at the respective health facility by trained nurses.

A total of seven questions, (1) Information about TB (2) Cause of TB (3) Major symptoms of TB (4) Transmissibility of TB (5) Mode of TB transmission (6) curability of TB, and (7) duration for TB treatment were asked to assess knowledge of the study participants about TB. Similarly, questions like information about HIV and its cause, its mode of transmission, treatment for HIV and preventive method for HIV infection were asked to assess knowledge of study participants about HIV. Questions like (1) Do you think there is any association between TB and HIV (2) Have you heard about HIV testing before you came here and 3) Do you feel that HIV testing is important for TB patients were asked to assess knowledge and perception of study participants about HIV testing. Questions like for how long have you experienced the current symptoms, fear of HIV testing can cause delay in treatment seeking and other reason (s) were asked to assess factors affecting treatment seeking of the study participants.

### Ethical consideration

The study protocol was reviewed and approved by Aklilu Lemma Institute of Pathobiology Institutional Review Board (IRB). The aim of the study was described to each study participant and oral informed consent was obtained from all participants prior to the commencement of the interview. To ensure confidentiality, study participants were represented by codes. The study participants were not interviewed about their HIV status. At the end of each interview, misconceptions were explained to the study participants.

### Data analysis

The collected data were computerized using Epi data version 3.1. Data cleaning was carried out by running frequency of each categorical variable and cross tabulation of different categorical variables. Descriptive statistics were summarized as percentage, means and standard deviations. The association of the outcome variables like knowledge of TB, knowledge of HIV, perception of PITC and treatment seeking with the socio-demographic characteristics of the participants was analyzed using logistic regression. The overall knowledge of TB, HIV, HIV testing and study participants’ treatment seeking behavior were assessed by scoring system. A score of one was given to correct responses, zero being used for incorrect/do not know responses. Mean value was used to categorize study participants in to two categories. Score less than the mean value was considered as low knowledge, while score greater than the mean value was considered as high knowledge [13]. Accordingly, study participants who scored above the mean value (3.95) of the seven questions for the knowledge of TB were categorized as knowledgeable. Similarly, the mean value (4.03) of the questions about knowledge of HIV was used to categorize the study participants in to two categories. Knowledge of HIV testing was categorized as low or high knowledge using mean value of 2.0. A mean value of 4.86 was used to categorized study participants into two categories (poor treatment seeking behavior or good treatment seeking behavior).

## Results

### Socio demographic characteristics of the study participants

A total of 415 study participants (age range from 16 to 60 years, mean age, 28.6 years) participated in the study. More than half (56.4%) of the study participants were Muslim and literate (52.5%). Table 1 shows the socio-demographic characteristics of the study participants.

**Table 1:** Socio-demographic characteristic of the study participants, Harar, Eastern Ethiopia, 2011

**Variable**		**Number**	**Percent**
Gender	Male	188	45.30
	Female	227	54.70
Age in years	<21	76	18.3
	21-40	296	71.4
	>40	43	10.6
Residence	Urban	171	41.2
	Rural	244	58.2
Religion	Christian	172	41.45
	Muslim	234	56.4
	Others	9	2.2
Marital status	Married	307	74
	Single	107	25.8
	Divorced	1	0.24
Educational status	Illiterate	197	47.5
	Primary	46	11.1
	Secondary	155	37.4
	Tertiary	17	4.1
Occupation	Unemployed	186	44.8
	Self employed	48	11.6
	Gov. employed	181	43.6
Distance from health institution	< 10 km	127	30.6
	>10km	288	69.4
Family size	1-3	312	75.2
	≥4	103	24.8
Forms of TB of study participants	PTB	376	90.6
	EPTB	39	9.4
Patients’ category	New	408	98.3
	Default	7	1.7

### Knowledge of study participants about TB

The majority (83.1%) of the study participants reported that they have heard about TB before coming to the respective health facility. However, the proportion of male study participants who heard about TB was significantly higher than that of the female study participants (89.4% vs 78%, X^2^= 9.5109, P= 0.002). About 24.3% of the study participants mentioned bacteria as a cause of the disease. About 77.3% of the study participants mentioned TB as a transmittable disease, while higher proportion of male study participants believed that TB is transmissible than female participants (83.0% vs 72.7%, X^2^ = 6.2166, P = 0.013). More than half (55.7%) of the respondents suggested that TB can be transmitted through droplets during sneezing and coughing. Relatively, higher proportion of male respondents mentioned the correct mode of transmission of TB than female respondents (61.2% vs 51.1%, X^2^= 4.2244, P = 0.040).

Relatively, a higher proportion of study participants in the age group < 21 years (31.6%) mentioned bacteria as the cause of TB compared to age group between 21-40 (25.7%) and > 40 years (2.3%) (p< 0.05). Higher proportion of study participants in the age group < 21 years (94.7%) responded that TB is a transmissible disease compared to age group between 21 and 40 years (73%) or over 40 years (76.7%) (X^2^= 16.3593, P < 0.001). Nearly two thirds (59%) of the study participants mentioned cough greater than 2 weeks, night sweat, weight loss and loss of appetite as the major symptoms of TB, whereas, about 72.5% of the study participants mentioned that TB treatment course takes 6 months.

Out of the 415 study participants, 277(66.8%) scored above mean regarding the overall knowledge of TB. Crude and adjusted effects of selected covariates obtained from logistic regression are summarized in Table 2 for the overall knowledge about TB. Age group over 40 years was significantly associated with overall knowledge of TB [adjusted odds ratio (AOR) = 4.19, 95% CI: 1.24 - 14.13, P=0.021]. Study participants from distance > 10 km had low knowledge about TB (AOR = 0.48, 95% CI: 0.24 - 0.97, P=0.042).

**Table 2:** Association of study participants’ socio demographic characteristics with their overall knowledge about HIV, Harar, Eastern Ethiopia, 2011

**Variable**		**Knowledge about TB**			
		**High** **n (%)**	**Low** **n (%)**	**COR** **(95% CI)**	**AOR** **(95% CI)**
Sex	Male	138 (73.4)	50 (26.6)	R	
	Female	139 (61.2)	88 (38.8)	0.57 (0.37 - 0.87)	1.33 (0.75 - 2.38)
Age	< 21 years	59 (77.6)	17 (22.4)	R	
	21-40 years	189 (63.9)	107 (36.1)	0.51 (0.28 - 0.91)	2.63 (0.99 -6.94)
	>40 years	29 (67.44)	14 (32.5)	0.60 (0.26 - 1.37)	4.19 (1.24 - 14.13)
Address	Urban	147 (85.9)	24 (14.0)	R	
	Rural	130 (53.3)	114 (46.7)	0.19 (0.11 - 0.31)	0.50 (0.24 - 1.06)
Religion	Orthodox	124 (72.1)	48 (27.9)	R	
	Muslim	144 ( 61.5)	90 (38.5)	0.62 (0.41 - 0.95)	0.97 (0.54 - 1.73)
	Others	9 (100.00)	0 (0.00)		
Marital status	Married	173 (56.35)	134 (43.6)	R	
	Single	103 (96.3)	4 (3.7)	19.9 (7.16 - 55.5)	34.58 (9.58-24.88)
	Divorced	1 (100.00)	0 (0.00)		
Distance from HI	≤ 10 km	99 (68.5)	28 (22.1)	R	
	>10 km	178 (61.8)	110 (38.2)	0.45 (0.28 - 0.74)	0.48 (0.24 - 0.97)

R: Reference value, HI: Health institution, COR: Crude odds ratio, AOR: Adjusted odds ratio, CI: Confidence interval.

### Knowledge of study participants about HIV

Out of the 415 study participants, 398 (95.9%) reported that they heard about HIV before coming to the health facility. However, the proportion of female study participants who heard about HIV was significantly higher than that of the male study participants (99.1% vs 92%, X^2^= 13.1865, P< 0.001). Higher proportion of individuals in the age group less than 21 years (98.7%) and age group between 21 and 40 years (98%) heard about HIV compared to individuals in the age group over 40 years (76.7%) (p < 0.05). Most (94.7%) of the study participants mentioned that HIV is transmitted through unsafe sexual intercourse with HIV infected individuals or needle stick injuries, contaminated blood and other body fluids. There was a significant difference between the proportion of males and females who suggested unsafe sexual intercourse as mode of transmission of HIV (90.4% vs 98.2%, X^2^= 12.5020, P< 0.001). On the other hand, higher proportion of individuals in the age group less than 21 years (97.7%) and age group between 21 and 40 years (96.3%) suggested unsafe sexual intercourse as the main mode of HIV transmission compared to individuals in the age group over 40 years (77.6%) (p < 0.05). Considerable number (189, 45.5%) of the respondents mentioned that a healthy looking person can be positive for HIV infection. However, higher proportion of males (51%) compared to females (34.4%) mentioned that a healthy looking person can be positive for HIV infection (p< 0.05). Similarly, higher proportion (67.1%) of individuals in the age group less than 21 years and in the age group between 21 and 40 years (43.2%) mentioned that a healthy looking person can be positive for HIV infection compared to individuals in the age groups over 40 years (23.3%) (p <0.05). The majority (96.6%) of the study participants responded that HIV can’t be cured and it has no treatment. About 30% of the study participants believed that there is an association between TB and HIV. Higher proportion of the respondents in the age group less than 21 years (46%) mentioned that there is an association between TB and HIV (i.e. TB is common in HIV infected person) compared to age group between 21 and 40 years (30.4%) and age group over 40 years (0%) (p< 0.05).

Out of the total 415 study participants, 202 (48.7%) scored above mean regarding overall knowledge about HIV. Crude and adjusted effects of selected covariates obtained from logistic regression are summarized in Table 3 for the overall knowledge about HIV. Muslim study participants were less likely to be knowledgeable about HIV compared to Orthodox (AOR =0.06, 95% CI: 0.03-0.11, P<0.001). Study participants from remote areas had better knowledge about HIV (AOR =2.55, 95% CI: 1.27-5.11,P= 0.008) compared to study participants from distance < 10 km from health facility.

**Table 3:** Association of study participants’ socio demographic characteristics with their overall knowledge about HIV

**Variable**		**Knowledge about HIV**			
		**Better knowledgeable (%)**	**Less knowledgeable (%)**	**COR (95% CI)**	**AOR (95% CI)**
Sex	Male	113 (60.1)	75 (39.9)	R	
	Female	89 (39.2)	138 (60.8)	0.42 (0.28 - 0.63)	0.76 (0.43 - 1.35)
Age	<21 years	52 (68.4)	24 (31.58)	R	
	21-40 years	140 (47.3)	156 (52.7)	0.41 (0.24 - 0.70)	0.32 (0.14 - 0.68)
	>40 years	10 (23.3)	33 (76.7)	0.13 (0.05 - 0.32)	0.68 (0.23 - 1.99)
Residence	Urban	112 (65.5)	59 (34.5)	R	
	Rural	90 (36.9)	154 (63.1)	0.30 (0.20 -0.46)	0.57 (0.28 - 1.15)
Religion	Orthodox	134 (77.9)	38 (22.1)	R	
	Muslim	60 (25.6)	174 (74.4)	0.09 (0.06 - 0.15)	0.06 (0.03 - 0.11)
	Others	8 (88.9)	1 (11.1)	2.26 (0.27 - 8.70)	0.92 (0.10 - 8.44)
Marital status	Married	123 (40.1)	184 (59.9)	R	
	Single	78 (72.9)	29 (27.1)	4.02 (2.48 - 6.52)	1.87 (0.79 - 4.43)
	Divorced	1 (100.0)	0 (0.00)		
Distance from HI	≤ 10 km	60 (47.2)	67 (52.8)	R	
	>10 km	142 (49.3)	146 (50.7)	1.08 (0.71 - 1.64)	2.55 (1.27 - 5.11)

R: Reference value, HI: Health institution, COR: Crude odds ratio,AOR: Adjusted odds ratio, CI: Confidence interval.

### Knowledge of study participants about HIV testing

The knowledge of the study participants about HIV testing is summarized in Table 4. The majority (81%) of the study participants reported that they had no information about PITC before they came to the respective health facility. Relatively, higher proportion of the study participants in the age group less than 21 years mentioned that they heard about HIV-testing compared to the other age groups ( X^2^= 9.0590, P = 0.011). Two thirds (66.5%) of the study participants believed that HIV testing is important for TB patients. However, higher proportion of males thought that HIV testing is important for TB patients than females (81.9% vs 53.7%, X^2^= 36.6348, P<0.001).

**Table 4:** Study participants’ knowledge about HIV testing, Harar, Eastern Ethiopia, 2011

**Variable**	**Knowledge about HIV testing**						
	**Gender**			**Age category**			
	**M (%)**	**F (%)**	**Total (%)**	**<21 (%)**	**21-40(%)**	**>40(%)**	**Total (%)**
Heard about HIV testing before?							
No	61 (32.5)	55 (24.2)	116 (28)	13 (17.1)	88 (29.7)	15 (34.9)	116 (28)
Yes	127 (67.5)	172 (75.8)	299 (72.1)	63 (82.9)*	208 (70.3)*	28 (65.1)*	299 (72)
Source of information Mass media, health personnel, Closed							
friends,	37 (29.1)*	86 (50)*	123 (41.1)	33 (52.4)*	90 (43.3)*	0 (0.00)	123 (41.1)
Other	90 (70.9)	86 (50)	176 (58.9)	30 (47.6)	118 (56.7)	28 (100)	176 (58.9)
Before you came here, do you know all TB patients will be tested for HIV?							
No	152 (80.9)	184 (81.1)	336 (81)	53 (69.7)	250 (84.5)	33 (76.7)	336 (81)
Yes	36 (19.1)	43 (18.9)	79 (19)	23 (30.3)*	46 (15.5)*	10 (23.3)*	79 (19)
Do you feel HIV test is important for TB patients?							
No	34 (18.1)	105 (46.3)	139 (33.5)	4 (5.3)	124 (41.9)	11 (25.6)	139 (33.5)
Yes	154 (81.9)*	122 (53.7)*	276 (66.5)	72 (94.7)*	172 (58.1)*	32 (74.4)*	276 (66.5)
When is HIV testing important?							
Every 3 months	54 (28.7)*	112 (49.3)*	166 (40)	40 (52.6)*	116 (39.2)*	10 (23.3)*	166 (40)
I don’t know	134 (71.3)	115 (50.7)	249 (60)	36 (47.4)	180 (61.8)	33 (76.7)	249 (60)

*significant difference between male and female and age groups (P <0.05).

Among the total 415 study participants, 181 (43.61%) scored above mean regarding overall knowledge about HIV testing. Single marital status of the study participants was significantly associated with better overall knowledge about HIV testing (AOR = 6.87, 95% CI: 3.00 - 15.71, P< 0.001). Study participants who came from remote areas were 0.12 times less likely to be knowledgeable about PITC (AOR = 0.12, 95% CI: 0.06 -0.23, P<0.001) (Table 5) compared to participants who came from < 10 km.

**Table 5:** Association of study participants’ socio demographic characteristics with their overall knowledge about HIV testing, Harar, Eastern Ethiopia, 2011

**Variable**		**Knowledge about HIV testing**			
		**High** **n (%)**	**Low** **n (%)**	**COR** **(95% CI)**	**AOR** **(95% CI)**
Sex	Male	101 (55.80)	87 (37.18)	R	
	Female	80 (44.20)	147 (62.82)	0.46 (0.31 - 0.69)	0.73 (0.40 - 1.34)
Age in years	<21 years	40 (17.1)	36 (19.9)	R	
	21-40 years	178 (76.1)	117 (64.6)	0.72 (0.43 - 1.20)	1.99 (0.96 -4.12)
	>40 years	16 (6.8)	28 (15.5)	2.07 (0.95 - 4.48)	5.55 (1.76 - 17.46)
Residence	Urban	113 (62.43)	58 (24.79)	R	
	Rural	68 (37.57)	176 (75.21)	0.19 (0.12 - 0.30)	0.71 (0.38 - 1.33)
Religion	Christian	90 (49.72)	82 (35.04)	R	
	Muslim	83 (45.86)	151 (64.53)	0.50 (0.33 - 0.74)	0.66 (0.38 - 1.15)
	Others	8 (4.42)	1 (0.43)	7.28 (0.89 - 59.53)	2.08 (0.23 - 8.68)
Marital status	Married	111 (61.33)	196 (83.76)	R	6.87 (3.00 - 15.71)
	Single	69 (38.12)	38 (16.24)	3.2 (2.02 - 5.07)	
	Divorced	1 (0.55)	0 (0.00)		
Distance from HI	≤10 km	94 (52)	33 (14.1)	R	
	>10 km	87 (48)	201 (85.9)	0.15 (0.09 - 0.24)	0.12 (0.06 - 0.23)

R: Reference value, HI: Health institution, COR: Crude odds ratio, AOR: Adjusted odds ratio, CI: Confidence interval.

### Treatment seeking behavior of study participants

Out of the 415 study participants, 199 (48%) reported that they visited the respective health facility after 6 weeks from the onset of their illness. However, higher proportion of female participants reported delaying for greater than 6 weeks compared to male participants (55.1% vs 39.4%, X^2^ = 10.1617, P < 0.001). Nearly two thirds (64.6%) of the study participants thought that fear of HIV-testing can delay treatment seeking of TB patients. The majority (81.6%) of the study participants in the age category less than 21 years claimed that fear of HIV testing can delay their treatment seeking compared to other age groups (X^2^ =12.8959, P = 0.002). About 12.8% of the study participants mentioned fear of HIV testing for their treatment delay. However, the majority (87.2%) mentioned reasons like they did not realize their illness as TB, lack of money and other social factors (Table 6).

**Table 6:** Study participants' treatment seeking behavior, Harar, Eastern Ethiopia, 2011

**Variable**	**Treatment seeking behavior**						
		**Gender**		**Age category**			
	**M (%)**	**F (%)**	**Total (%)**	**<21 (%)**	**21-40(%)**	**>40 (%)**	**Total (%)**
For how long did you have these symptoms?							
≤ 6 weeks	114 (60.6)*	102 (45)*	216 (52)	33 (43.4)*	150 (50.7)*	33 (76.7)*	216 (52)
> 6 weeks	74 (39.4)	125 (55)	199 (48)	43 (56.6)	146 (49.3)	10 (23.3)	199 (48)
HIV testing has impact on the treatment seeking behavior?							
No	62 (33)	85 (37.4)	147 (35.4)	14 (18.4)	113 (38.2)	20 (46.5)	147 (35.4)
Yes	126 (67)	142 (62.6)	268 (64.6)	62 (81.6)*	183 (61.8)*	23 (53.5)*	268 (64.6)
Reason for your delay Lack of money, disease awareness	155 (82.5)	207 (91.2)	362 (87.2)	57 (75)	267 (90.2)	38 (88.4)	362 (87.2)
Fear of HIV testing	33 (17.5)*	20 (8.8)*	53 (12.8)	19 (25)*	29 (9.8)*	5 (11.6)*	53 (12.8)
First treatment as a relief							
Drug venders or traditional healers	120 (63.8)	130 (57.3)	250 (60.2)	60 (79)	162 (54.7)	28 (65.1)	250 (60.2)
Health institutions	68 (36.2)*	97 (42.7)*	165 (39.8)	16 (21)*	134 (45.3)*	15 (34.9)*	165 (39.8)

*Significant difference between male and female and age groups (P <0.05).

Among the total 415 study participants, 212 (51.1%) scored above mean concerning good treatment seeking behavior. Being female study participants (AOR = 0.11, 95% CI: 0.05-0.25, P<0.001), rural resident (AOR = 0.002, 95% CI: 0.00-0.01, P<0.001), single marital status (AOR =0.001, 95% CI: 0.00 - 0.01, P= 0.000) and distance > 10 km (AOR =0.46, 95% CI: 0.28 - 0.75, P=0.002) were associated with low treatment seeking of the participants. Knowledge of study participants about HIV testing (AOR =206, 95% CI: 48.58 - 876.13, P<0.001) was significantly associated with high treatment seeking behavior (Table 7).

**Table 7:** Association of study participants’ socio demographic characteristics with their treatment seeking behavior, Harar, Eastern Ethiopia, 2011

**Variable**		**Treatment seeking behavior (TSB)**			
		**High** **n(%)**	**Low** **n (%)**	**COR** **(95% CI)**	**AOR** **(95% CI)**
Sex	Male	121(64.4)	67 (35.6)	R	R
	Female	91 (40.1)	136 (59.9)	0.37 (0.24 - 0.55)	0.11 (0.05 - 0.25)
Age in years	<21 years	36 (47.4)	40 (52.6)	R	R
	21-40 years	143 (48.3)	153 (51.7)	1.03 (0.62 - 1.72)	3.03 (0.57 - 16.03)
	>40 years	33 (76.7)	10 (23.3)	3.66 (1.58 - 8.48)	1.31 (0.18 - 9.43)
Residence	Urban	124 (72.5)	47 (27.5)	R	R
	Rural	88 (36.1)	156 (63.9)	0.21 (0.13 - 0.32)	0.002 (0.00 - 0.01)
Religion	Orthodox	92 (53.5)	80 (46.5)	R	R
	Muslim	113 (48.3)	121 (51.7)	0.81 (0.54 - 1.20)	20 (3.61 - 111.70)
	Others	7 (77.8)	2 (22.2)	3.04 (0.61 - 15.07)	0.80 (0.04 - 14.09)
Marital status	Married	151 (49.2)	156 (50.8)	R	R
	Single	61 (57.0)	46 (43)	1.36 (0.87 - 2.13)	0.001 (0.00 - 0.01)
	Divorced	0 (0.00)	1 (100)		
Distance from HI	≤10 km	91 (71.6)	36 (28.4)	R	R
	>10km	121 (42.0)	167 (58.0)	0.28 (0.18 - 0.45)	0.46 (0.28 - 0.75)
Knowledge of TB	Low Knowledge	33 (23.9)	105 (76.1)	R	R
	High Knowledge	179 (64.6)	98 (35.4)	5.81 (3.66- 9.22)	1.91 (0.94 - 3.87)
Knowledge of HIV	Low Knowledge	79 (37.1)	134 (62.9)	R	R
	High Knowledge	133 (65.8)	69 (34.2)	3.26 (2.18 - 4.88)	1.82 (0.34 - 9.72)
Knowledge HIV testing	Low Knowledge	58 (24.8)	176 (75.2)	R	R
	High Knowledge	154 (85.1)	27 (14.9)	1.7 (10.44 - 28.68 )	2.06 (4.58 -8.76)
Reason for your treatment delay	Lack of money or				
	awareness	177 (48.9)	185 (51.1)	R	R
	Fear of HIV testing	35 (66.0)	18 (34.0)	2.03 (1.11 - 3.72)	1.66 (0.72 - 3.81)

## Discussion

This study demonstrates that there is a wide knowledge gap regarding TB among the study participants. Majority of the study participants didn’t know bacteria as the cause of TB. They mentioned wind, cold and spiritual punishment as the cause of the disease which is similar to findings of other studies from other parts of Ethiopia [13,14]. Misconception about the correct cause of the disease could affect patient attitude towards health-seeking behavior and preventive methods. On the other hand, study participants had good knowledge of the mode of transmission of TB its signs and symptoms which is better than the findings of study conducted in Jimma [14] and lower than the findings of study conducted in Afar [13]. In the present study, distance > 10 km from the health facilities was found to be one of the factors that associated with low knowledge of the participants about TB. This finding supports the findings of study conducted in Tigray, which showed that study participants from long distance had low knowledge about TB [15]. This might be due to less access to media or low educational status of rural residents.

In this study, about half of the study participants (51.3%) had low overall knowledge about HIV which is inconsistent with the findings of study from the northwest of Ethiopia [16]. The majority of the study participants had also low awareness about the association between TB and HIV infection when compared to the results of other study conducted in Ethiopia [11]. This situation could affect the implementation of the PITC program in the present study area. Hence, there is a need to consider potential determinants for the acceptance of HIV testing among the TB patients.

As voluntary counselling and testing services are important for slowing down of HIV spread, there is a need to consider potential determinants for the acceptance of HIV testing among the TB patients. In this study, we assessed potential factors like socio-demographic characteristics that might have impact on the knowledge about HIV testing in the present study area. The overall study participants’ knowledge about HIV testing was low compared to the findings of studies conducted in other parts of Ethiopia [16,17]. The findings of this study also revealed that older aged and study participants from a long distance had low level of knowledge about HIV testing, which is comparable to the findings of studies in other parts of Ethiopia [16,18]. Hence, public education on HIV testing should focus on older age groups and individuals from distance > 10 km from health facility in the present study area.

Understanding TB patients’ treatment seeking behavior and factors influencing the choice of healthcare is particularly important for evidence-based TB control/interventions. In this study, an attempt was made to explore the fear of HIV testing on the treatment seeking behavior of TB patients. The results revealed that more than half of the participants (64.6%) thought that fear of PITC could cause delay in the treatment seeking of TB patients. Moreover, the majority (81.6%) of the study participants in the age category less than 21 years claimed that HIV testing has impact on their treatment seeking behavior, because of fear of HIV serostatus and the related stigma and discrimination as reported from elsewhere [19-21]. The national TB and HIV control guideline in Ethiopia recommends HIV counselling and testing as a routine care for all TB patients to reduce TB-HIV related morbidity and mortality [10]. In order to get the desired results in TB-HIV control efforts, community based education of TB-HIV co-infection would be important in addition to health facility based educating individual patient.

## Conclusion

The findings of this study revealed that the study participants had low knowledge about TB-HIV co-infection and HIV testing. This necessities for using best health education approach which improves knowledge of TB- HIV co-infection and HIV testing to get the desired results in TB-HIV control efforts. Age dependent fear of HIV testing was also observed among the study participants age less than 21 years. Therefore, attention needs to be given to improve fear of HIV testing in the age group less than 21 years in the present study area. Further investigation about the impact of fear of HIV testing on the treatment seeking behavior of TB is vital in other parts of Ethiopia for sustainability and effectiveness of the PITC program in the country.

## Competing interests

The authors declare that they have no competing interests.

## Authors' contributions

AS designed the study, participated in data collection, analysis and drafted the manuscript. ML, participated in study design, analysis, write-up and critically revised the manuscript. Both authors read and approved the final manuscript. ML is the guarantor of the paper.

## Pre-publication history

The pre-publication history for this paper can be accessed here:

http://www.biomedcentral.com/1471-2458/13/124/prepub
